# Communication Skills Training in Veterinary Education: A Scoping Review of Programs and Practices

**DOI:** 10.3390/vetsci13010063

**Published:** 2026-01-09

**Authors:** Verónica López-López, Montserrat Poblete Hormazábal, Sergio Cofré González, Constanza Sepúlveda Pérez, Carolina Muñoz Pérez, Rafael Zapata Lamana

**Affiliations:** 1 Departamento de Ciencias Clínicas, Facultad de Ciencias Veterinarias, Universidad de Concepción, Oficina de Educación Médica Veterinaria, Chillán 3812120, Chile; veronicalopez@udec.cl (V.L.-L.); montsepoblete@udec.cl (M.P.H.); 2 Departamento de Ciencias Clínicas, Facultad de Ciencias Veterinarias, Universidad de Concepción, Chillán 3812120, Chile; sercofre@udec.cl; 3 Oficina de Educación Médica Veterinaria, Facultad de Ciencias Veterinarias, Universidad de Concepción, Chillán 3812120, Chile; conssepulveda@udec.cl; 4 Escuela de Educación, Universidad de Concepción, Los Ángeles 4451032, Chile; caromunozp@udec.cl; 5 Escuela de Kinesiología, Facultad de Salud, Universidad Santo Tomás, Los Ángeles 4441171, Chile

**Keywords:** clinical communication, veterinary medicine, educational programs, communication skills

## Abstract

Background: Effective communication is a fundamental competency in veterinary medicine that shapes the quality of veterinarian–client relationships, shared decision-making, and animal welfare. However, consistent and systematic integration of communication training across veterinary curricula remains uneven worldwide. Methods: This scoping review mapped and analyzed educational programs aimed at developing communication competencies in veterinary education and professional practices. A systematic search was conducted according to PRISMA-ScR guidelines, identifying 37 eligible studies published between 2005 and 2024. Results: Most publications were in English and originated from North America, particularly Canada (*n* = 15) and the United States (*n* = 8). Regarding target populations, 15 studies (40.5%) focused on veterinary students, 12 (32.4%) on practicing veterinarians, 8 (21.6%) on animal owners or clients, and 2 on veterinary educators. 18 studies (48.7%) described structured programs that used active learning strategies such as role-play, clinical simulations, peer-assisted learning, and formative feedback. The competencies frequently emphasized include empathy, active listening, nonverbal communication, conflict resolution, and rapport building. Notable best practices included the Calgary–Cambridge model, Objective Structured Clinical Examination (OSCE), and reflective video analysis. Conclusions: The available evidence indicates a growing emphasis on clinical communication within veterinary education, primarily implemented through experiential and practice-based approaches. However, substantial gaps persist in the representation of Latin American contexts and in the systematic, longitudinal integration of communication skills across veterinary curricula. Addressing these gaps may contribute to more coherent, equitable, and context-sensitive communication training in veterinary education.

## Simple Summary

Effective communication is a key skill for veterinarians because it influences animal welfare, client satisfaction, and clinical decision-making. This scoping review analyzed 37 educational studies that investigated communication skills training in veterinary education and professional practice. Most studies originated from North America, primarily Canada (*n* = 15) and the United States (*n* = 8), while other regions, including Latin America, were underrepresented. Regarding target groups, 15 studies focused on veterinary students, 12 on practicing veterinarians, 8 on animal owners or clients, and 2 on veterinary educators. The reported educational approaches mainly involved structured training sessions that integrated theoretical lessons with practical exercises, role-playing to recreate clinical situations and provide feedback, experiential learning to encourage reflection based on personal experience, and peer-assisted learning to promote peer evaluation, practice among equals, and skill development in a collaborative environment. Addressing geographic disparities and strengthening faculty development are essential steps toward fostering more equitable and effective veterinary communication training worldwide.

## 1. Introduction

Effective communication is a fundamental competency in veterinary medicine, as it directly impacts the quality of treatment, well-being of patients, and the building of trust-based relationships with caregivers [1]. This skill encompasses not only the accurate transmission of information but also the emotional sensitivity required in delicate situations such as delivering bad news [2]. It is defined as the ability to know what to say, to whom, when, and how to express it, integrating linguistic, psychological, and sociological dimensions [3]. An effective communicator listens attentively, avoids interrupting, does not monopolize conversation, and uses appropriate and respectful language [4].

In both human and veterinary medicine, communication is recognized as a set of skills that optimizes patient care [5]. In veterinary practice, these competencies are essential, as they influence the accuracy of diagnoses, efficiency of treatments, and overall experience of caregivers and animals [1,6]. Both students and professionals agree on the importance of verbal communication and interpersonal skills in future careers [7]. However, deficiencies in this area are among the most frequent complaints in companion animal veterinary clinics [7].

Various factors influence the quality of veterinarian–caregiver interaction, including verbal and non-verbal language, the context of the consultation, previous experiences, and cultural environment. The absence of empathy can be a critical obstacle to effective communication [8]. For this reason, these competencies should be considered essential clinical skills in professional training [9].

In medical education, communication skills are integrated into both formal and hidden curricula, where instructors model behaviors [10]. In veterinary medicine, effective communication is associated with more accurate diagnoses, efficient time management, higher client satisfaction, and collaborative relationships between professionals and caregivers [10,11,12]. Furthermore, a brief but effective initial interaction can be decisive in establishing trust and leaving a positive impression [1].

Strengthening these competencies becomes especially important in sensitive contexts such as delivering bad news, where clarity, empathy, and emotional support are essential [2]. In this field, structured protocols such as Calgary–Cambridge [6] and EPICEE (SPIKES in Spanish) [13] have proven useful in guiding interviews through the following key stages: preparing the environment, exploring previous knowledge, tailoring information, managing emotions, and jointly planning future strategies.

Communication skills in veterinary medicine have been taught through active methodologies such as role-playing, simulations, peer learning, and formative feedback [3,10,11]. These activities allow progressive practice in safe environments before interacting with real patients, thus fostering the internalization of professional behaviors. The use of video recordings and structured assessments, such as the OSCE, has become an established tool for critical reflection and objective measurement of these competencies [5,10]. The early and systematic incorporation of these skills into the curriculum, together with direct observation and constructive feedback, is considered essential to ensure meaningful learning [9,11].

These gaps make it difficult to identify which competencies are most consistently addressed, which pedagogical combinations show the best cost–benefit ratios in different contexts, and which instruments offer the greatest utility and comparability for curricular decisions. Consequently, teaching teams and academic authorities lack an integrative map that articulates competencies, teaching strategies, assessment instruments, and outcomes while also highlighting research and implementation priorities for different scenarios (small animals, production, equine, undergraduate, postgraduate, and continuing education).

Given this situation, a scoping review is more appropriate than a classic systematic review, with the purpose of mapping the field, describing populations, concepts, and contexts (PCC), summarizing how communication is taught and assessed, and identifying gaps without seeking to estimate aggregate effects. This approach facilitates a broad synthesis useful for curriculum redesign, faculty development, and planning new evaluations and studies [14].

The main objective of this review was to comprehensively map the existing literature on communication competencies in veterinary education and professional practice. Specifically, this review seeks to (1) identify articles published in scientific journals focused on the implementation of educational programs in veterinary training; (2) classify articles published in scientific journals oriented toward the development of communicative competencies, considering year, country/language, level of training, type of participants, sample size, competencies to be developed, pedagogical strategy, dosage/duration, quantitative/qualitative results, barriers/facilitators, and limitations; and (3) describe the contribution of best practices and methodologies presented in scientific articles aimed at the implementation of educational programs in veterinary training. Through this systematic mapping, we aim to answer the following research questions: (1) What evidence is presented by published studies that have addressed the development of communicative competencies in the training of veterinarians, and what is their contribution to this development? (2) What recommendations do the studies suggest for developing communicative competencies in veterinary medicine? This review seeks to develop practical recommendations and propose a research agenda that can be applied in various academic and clinical settings in the field of veterinary medicine.

## 2. Materials and Methods

### 2.1. Design, Protocol, and Registration

A scoping review was conducted in accordance with the PRISMA-ScR. The PCC framework (Population, Concept, and Context) was applied to define the questions and guide the methodological decisions.

### 2.2. Eligibility Criteria (PCC)

- Population: veterinary medicine students, practicing veterinarians, and faculty/tutors; the participation of clients/owners as evaluators is accepted.
- Concept: Training and/or assessment of communication competencies (e.g., empathy, active listening, nonverbal communication, delivering bad news, and shared decision-making).
- Context: Clinical education/training at undergraduate, postgraduate, and continuing education levels in small animals, production animals, equines, and other settings.
- Language: articles in English, Portuguese, or Spanish with full texts available.
- Period: Publications between 2005 and April 2024 (inclusive).
- Document type: empirical studies, intervention/implementation descriptions, or educational assessments with sufficient data for extraction.
- Access and sufficiency: reports with a minimum level of detail on intervention and evaluation that allow for the extraction of predefined variables.

#### Exclusion Criteria

- Non-veterinary population or outside the clinical veterinary field.
- Not related to clinical veterinary communication (e.g., scientific/academic communication, outreach without a clinical educational component).
- Language other than English/Spanish without officially accessible full-text translations.
- Period outside this range (before 2005 or after April 2024).
- Ineligible document types: Editorials, letters, comments, protocols without results, books, and opinions without data.
- Insufficient information for the extraction of key variables, even after reasonable attempts to retrieve them.

### 2.3. Sources of Information and Coverage

ScienceDirect, PubMed, SciELO, and Scopus were searched. The coverage applied was 2005–April 2024, and English/Spanish were considered (according to the eligibility criteria established in Section 2.2).

### 2.4. Search Strategy

We defined two conceptual pairs and combined them using Boolean operators while maintaining precedence AND > OR as follows:

Pair A: “veterinary medicine” AND “educational program”; Pair B: “communication skills” AND “veterinary education”.

The base string was:

((“veterinary medicine” AND “educational program”) OR (“communication skills” AND “veterinary education”))

We applied filters according to language (English/Spanish), period (2005–April 2024), and document type (articles and reviews).

### 2.5. Selection of Sources of Evidence

First, deduplication was performed. Next, two reviewers independently screened the titles and abstracts, and subsequently the full text. Discrepancies were resolved by consensus or by the involvement of a third reviewer.

### 2.6. Data Extraction (Data Charting) and Variables

Data extraction was performed using a standardized spreadsheet. The following variables were recorded: year, country/language, level of education, type of participants, sample size, competencies to be developed, pedagogical strategy, dose/duration, quantitative/qualitative results, barriers/facilitators and limitations.

## 3. Results

Narrative and tabular syntheses were conducted without a meta-analysis. The studies were organized according to the PCC framework and presented as (i) selection flow diagram, (ii) general characteristics (year, language/country, population/role addressed), and (iii) pedagogical strategies (including assessment instruments). Where appropriate, the tables and figures accompany the text to facilitate reading.

### 3.1. Results of Selection Process 

**Figure 1:**
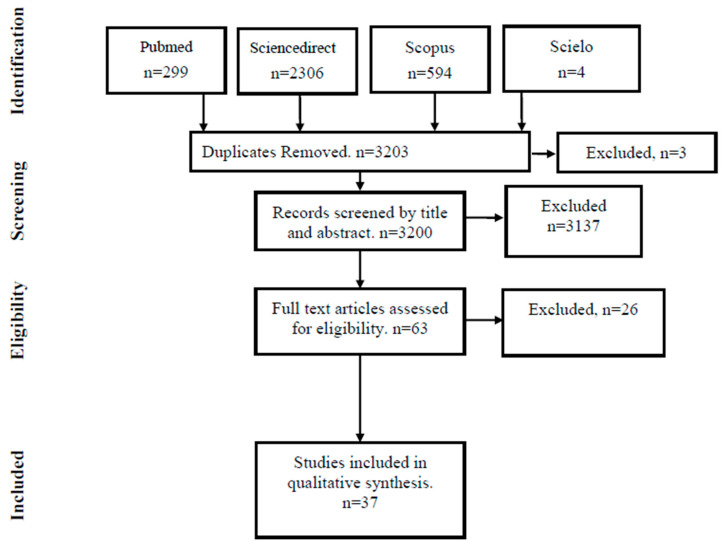
PRISMA-ScR diagram.

A total of 3203 records were identified in the databases (ScienceDirect, 2306; PubMed, 299; SciELO, 4; Scopus, 594). After removing duplicates (*n* = 3), 3200 records remained for title/abstract screening, of which 3137 were excluded because they did not meet the eligibility criteria. Sixty-three reports were retrieved (*n* = 0 not retrieved). Sixty-three full-text reports were assessed, of which twenty-six were excluded for documented reasons. A total of thirty-seven sources of evidence were included in the synthesis (Figure 1 PRISMA-ScR diagram).

### 3.2. Characteristics of the Included Studies

The 37 studies covered the period from 2005 to 2024 and mainly included reports in English, with a predominance of undergraduate student experience. The most frequent population was students, followed by professors and practicing veterinary doctors. The selected studies were mostly quantitative and mixed in design, with varying sample sizes and settings. The selected articles were classified into three key dimensions: language of publication (Section 3.2.1), geographic origin (Section 3.2.2), and population distribution (Section 3.2.3).

#### 3.2.1. Publication Language

Regarding the language of publication, there was an exclusive concentration of studies in English (100%), with no publications in Spanish or Portuguese within the analyzed period.

#### 3.2.2. Geographic Origin of the Studies

**Figure 2:**
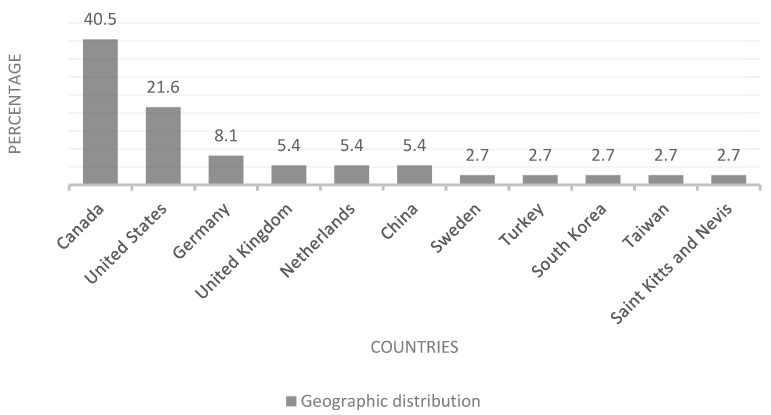
Geographic Origin of the Studies.

Of the 37 articles analyzed, there was a marked concentration of publications from English-speaking countries and the Northern Hemisphere. Figure 2 shows that most of the articles came from Canada (40.5%) [1,11,15,16,17,18,19,20,21,22,23,24,25,26,27] and the United States (21.6%) [28,29,30,31,32,33,34,35], which together accounted for more than 60% of the total. Other countries that contributed publications to the analysis included Germany (8.1%) [36,37,38], the United Kingdom (5.4%) [39,40], the Netherlands (5.4%) [41,42], China (5.4%) [7,43], and, to a lesser extent, Sweden [44], Turkey [45], South Korea [46], Taiwan [47], and Saint Kitts and Nevis [48], each with a 2.7% representation (Figure 2 Geographic origin of the studies).

#### 3.2.3. Distribution by Population/Role Addressed

**Figure 3:**
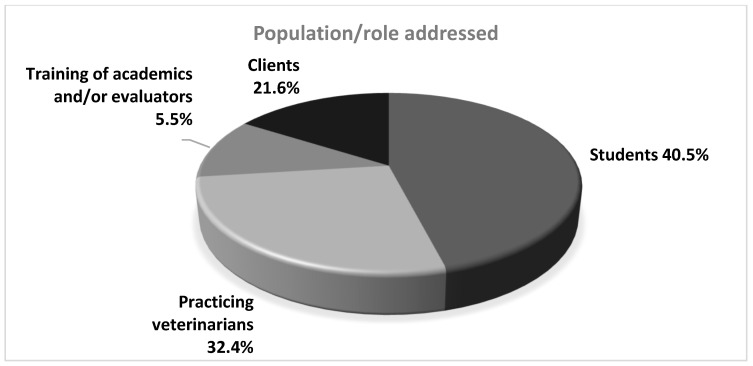
Distribution by population/role addressed.

This exploratory review made it possible to identify the various populations and roles addressed in the selected articles, providing a more comprehensive perspective on current priorities and methodological approaches for teaching communication skills in veterinary medicine. Of the 37 articles analyzed, 40.5% [11,22,23,25,26,27,28,29,31,32,33,34,35,45,46] focused on veterinary students, demonstrating a predominant interest in strengthening these skills in the early stages of training. In contrast, 32.4% [7,15,18,19,21,24,38,39,40,42,43,44] of the studies targeted practicing veterinarians, reflecting the need to implement continuing education strategies that allow for the updating and improvement of these skills in actual clinical settings. Similarly, 21.6% [1,16,17,30,36,37,41,47] of the articles addressed the satisfaction or perception of pet owners, positioning clients as key figures in evaluating the communication performance of veterinary professionals. Finally, 5.5% [20,48] of the studies focused on training academics and assessors in communication skills, highlighting the importance of having properly trained and up-to-date educators in this area to ensure effective teaching (Figure 3, Distribution by Addressed Population/Roles).

### 3.3. Implemented Educational Programs

Based on the declared pedagogical strategies, the analysis determined that 18 studies (48.7%) [7,11,18,20,21,22,23,25,26,28,29,33,35,40,43,44,45,48] addressed educational programs specifically implemented to develop communication skills in veterinary training. These include training experiences with a curricular structure, defined objectives, content selection, pedagogical activities, and explicit assessment strategies. These programs were applied both at the undergraduate level and in continuing professional development (CPD) for veterinarians, as well as in the training of instructors in communication skills.

Unlike conceptual studies or needs assessments, the 18 identified studies demonstrated explicit pedagogical intent, with a practical and assessable focus. This represents a significant advance in the field, as it shows that a considerable portion of the literature not only reflects the importance of communication in veterinary medicine but also implements concrete training proposals for its systematic development.

#### Identified Strategies

The analysis of the reviewed studies revealed a consistent emphasis on active strategies designed to enhance communication skills in veterinary education. Among these, structured feedback emerged as a central approach, allowing students to refine their interpersonal and clinical communication through targeted guidance. Reflective practice—often supported by video recordings of real or simulated interactions with tutors or clients—was also widely described as an effective tool for promoting self-awareness and professional growth. Many studies highlighted the application of the Calgary–Cambridge model as a pedagogical framework to structure clinical interviews and to guide both teaching and feedback processes. Communication performance was frequently evaluated in simulated settings, such as Objective Structured Clinical Examination (OSCE), providing a standardized and controlled environment for assessment. Role-play activities were commonly used as deliberate practice to rehearse clinical interactions and to consolidate feedback-driven learning. Additionally, several studies explored the teaching of “breaking bad news” through structured frameworks such as SPIKES or EPICEE, emphasizing the importance of empathy and emotional support in challenging conversations. Collectively, these strategies foster active learning and the acquisition of essential communication competencies, including active listening, empathy, clarity of information delivery, and the effective management of difficult situations.

The most reported strategies and resources are summarized below, along with their main formative functions.

- Structured feedback: A training tool used to develop the ability to provide and receive specific feedback based on observable behaviors, contributing to the continuous improvement of communication skills.
- Reflective practice with recordings: The use of videos (real or simulated cases) to observe, self-assess, and co-assess performance; identifying patterns; and opportunities for improvement.
- The Calgary–Cambridge model: A reference framework to structure the clinical interview and guide teaching and feedback; it is often implemented in small groups, which facilitates deliberate practice and guided discussion.
- Assessment in simulated environments/OSCE: Standardized stations are used to systematically assess communication performance and identify strengths and areas for improvement.
- Role-play: Rehearsal of clinical interviews with simulated tutors/clients with immediate and guided feedback; it promotes deliberate practice, management of difficult situations, and adjustment of verbal/non-verbal communication.

Based on these highlighted strategies and resources, Table 1 systematizes their operational descriptions, pedagogical functions, and implementation recommendations, indicating the studies that support them.

## 4. Discussion

A key finding of this exploratory review is the marked linguistic concentration in English (100%), along with the predominance of publications from the Northern Hemisphere, particularly Canada (40.5%) and the United States (21.6%). This high representation can be explained by the advanced development of veterinary education in these countries, as well as by their well-established tradition in educational research. The lack of studies in Spanish or Portuguese reflects not only the limited accessibility of literature for Latin American academic communities but also the scarce representation of diverse cultural contexts in the pedagogical development of veterinary medicine in Latin America.

This result highlights a significant barrier to access to scientific knowledge for teachers, researchers, and students who do not speak English and reveals the lack of representation of Latin American educational contexts in the international literature on communication competencies in veterinary medicine. Similar gaps may also affect other underrepresented geographical and linguistic contexts, where limited research visibility and contextual constraints restrict the dissemination of educational initiatives in veterinary communication. In this regard, there are still relevant areas to be developed in Latin America, such as the systematization of training experiences in clinical communication, conducting studies that consider the local cultural and linguistic context, and evaluating pedagogical methodologies adapted to the educational realities of the region. Overcoming these gaps will require strengthening regional research networks, promoting scientific publications in languages accessible to teachers and students, and fostering educational policies that formally integrate communication skills into veterinary school curricula. Advances in these areas will provide greater visibility to Latin American experiences and enrich veterinary education with more diverse and contextualized approaches.

This gap presents an opportunity to promote the internationalization of knowledge by strengthening multilingual scientific production and interregional collaboration.

Regarding the target population, 40.5% of the articles focused on veterinary students, while the remainder were distributed among practicing professionals, teachers and tutors. This trend reveals a focus on initial training, with little attention to a comprehensive and longitudinal approach. Communication competencies should be considered not only as an undergraduate objective but also as a transversal and ongoing skill in clinical practice.

The findings of this review support the notion that the development of communication competencies has a direct impact on the quality of care, relationship with caregivers, and clinical effectiveness, although historically, these have been given lower priority than technical skills [10,11]. Multiple studies agree that effective communication improves the caregiver’s experience, optimizes understanding of treatment, reduces legal risks, and strengthens trust in the professional [1,7]. However, there continues to be wide variability among institutions regarding their requirements, methodology, and assessments. While some programs are limited to theoretical courses, others implement active strategies, such as clinical simulations, role-playing, case analysis, and structured feedback, in line with [5,11]. While North America and Europe have advanced in this field, systematic applications remain limited in other contexts [3,8]. Black, Latino, Asian, Native Hawaiian, and Pacific Islander populations are considered underrepresented in veterinary medicine [49].

Teaching specific skills, such as delivering bad news, has become crucial. Models such as Calgary–Cambridge [6] and EPICEE (SPIKES) [13] offer structured frameworks to address highly complex situations in which empathy, emotional control, and clear information are essential [2]. The literature suggests that the most successful approaches are those that progressively integrate these competencies from the early years of education through supervised clinical practice [3].

Experiential methodologies, such as role-playing, video recording, and interaction simulation, have proven highly effective in facilitating critical reflection and immediate feedback [6]. However, factors such as teacher training, resource availability, and institutional culture play a decisive role in their implementation. In this regard, the role of the teaching staff should not be limited to the transmission of knowledge but should also incorporate the modeling of communicative behaviors [3]. The small number of studies focusing on teacher training (5.5%) confirms that this dimension requires further development.

Another underrepresented aspect is the inclusion of tutors as active agents in the training process. Only 21.6% of studies included their perspectives, despite being central to the clinical dynamic. As noted by [7], integrating the client’s perspective in the assessment of communication competencies would improve curricular relevance and support a more tutor- and patient-centered approach to clinical practice.

Consequently, it is necessary to move towards a broader conceptualization of veterinary communication that transcends the transfer of information and encompasses emotional, cultural, and ethical dimensions [8]. Incorporating these competencies as a cross-cutting axis within the curriculum would lead to more ethical, critical, and humanized training, especially in delicate situations such as grief management or communicating bad news.

Finally, the findings highlight the need to promote research in Latin America, where the validation of native and culturally relevant models is a priority. Training in communication should be understood as a continuous and progressive process that begins in the early years of education, is systematically assessed, and remains a priority throughout one’s professional life.

## 5. Conclusions

This scoping review found a diverse range of studies that highlight communication as an essential competency in veterinary education and professional practice. These studies primarily focus on structured educational interventions and experiential learning strategies. While the findings show a consistent emphasis on developing communication skills in specific contexts, there is a need for longitudinal and comparative studies to investigate their recognition at academic institutions, integration into curricula, and development over time. This is particularly important in regions that are currently underrepresented, such as Latin America.

The studies analyzed showed a trend towards the use of active and experiential strategies focused on key competencies such as empathy, active listening, and nonverbal communication. In this context, the Calgary–Cambridge model, simulated environments/OSCE (as an assessment tool), and formative feedback are systematically considered key resources for instructional design and evaluation of communicative performance. Likewise, role-playing, clinical simulations, recordings for reflective practice, and early integration into the curriculum provide an operational basis for strengthening teaching–learning programs.

Looking ahead, the priorities are as follows:

- Faculty training and modeling communicative behaviors;
- Inclusion of tutors/clients as formative actors and, where appropriate, as evaluators;
- Development and validation of contextualized models that consider cultural and resource-specific particularities;
- Curricular integration of communication with systematic assessment (e.g., rubrics, OSCE) and longitudinal monitoring.

With these emphases, communication can be consolidated as a cross-cutting pillar in veterinary education, with the potential to impact the quality of care, relationship with tutors/clients, and clinical effectiveness across diverse contexts.

## Figures and Tables

**Table 1:** Educational Interventions/Strategies to Strengthen Communicative Competencies.

Included Studies (ID)	Educational Intervention/ Strategy	Operational Description	Main Pedagogical Function	Implementation Recommendations (Context)
[7,18,21,22,25,26,28,33,40,45]	Structured feedback	Specific feedback based on observable behavior.	Continuous improvement and communication safety through deliberate practice of receiving feedback.	Teach feedback from early stages; use rubrics/brief guides after simulations or practice sessions.
[11,25,35]	Reflective video practice	Use of videos (real or simulated cases) for observation, self-assessment, and peer evaluation.	Identification of patterns and areas for improvement: Active and reflective learning.	Implement practical classes and guided reflection sessions; assign pre-/post-review tasks.
[11,20,21,25,40,43]	Calgary–Cambridge Model	Framework for structuring the clinical communicative interview and guiding teaching/feedback.	Organization of interaction, improved relationships with tutors/clients, and communicative effectiveness.	Workshops and small groups; mastery sequences; practice with checklists.
[18,23,48]	Simulated settings/OSCE	Standardized stations for the systematic assessment of communicative performance.	Objective detection of strengths and areas for improvement.	Integration into formal evaluations and clinical simulations.
[28,44]	Role-playing games (role-play)	Rehearsal of interviews with simulated clients and immediate feedback.	Deliberate practice and handling of difficult situations.	Integrate into short workshops/small groups; use behavior rubrics.

Abbreviations: OSCE, Objective Structured Clinical Examination. The table summarizes the key active-learning strategies identified in the 18 studies that implemented educational programs, highlighting their operational characteristics, pedagogical purposes, and recommended implementation contexts.

## Data Availability

No new data were created or analyzed in this study. Data sharing is not applicable to this article.

## Abbreviations

The following abbreviations are used in this manuscript:

CPD	Continuing Professional Development
OSCE	Objective Structured Clinical Examination
PCC	Population–Concept–Context
PRISMA-ScR	Preferred Reporting Items for Systematic Reviews and Meta-Analyses—Scoping Review extension
EPICE	Entorno, Percepción, Información, Comprensión, Emoción y Estrategia (Spanish adaptation of the SPIKES protocol for delivering bad news)
SPIKES	Setting, Perception, Invitation, Knowledge, Emotions, and Strategy
